# Multi-Study Coordinated Analysis to Promote Replicability: Example Using Self-Rated Health

**DOI:** 10.1093/geroni/igaf122.1459

**Published:** 2025-12-31

**Authors:** Daniel Mroczek

**Affiliations:** Northwestern University, Evanston, Illinois, United States

## Abstract

Self-reported health is a non-invasive and low-cost way of identifying who is at risk of poor health in later life. We examined the extent to which self-rated health was associated with cognitive health outcomes, both cross-sectionally and longitudinally. Using the coordinated data analysis methodological framework to promote robustness an replicability in our findings, we identified 14 longitudinal datasets worldwide that met analytic inclusion criteria. Our pre-registered analyses tested whether self-rated health was associated with cognitive function both concurrently and at longitudinal follow up, as well as with odds of developing cognitive impairment or dementia over the course of the study, net of demographic and health covariates. Results indicated that, across nearly all datasets, higher self-rated health was associated with better cognitive function (both concurrently and longitudinally) and lower odds of developing dementia, over and above the effects of objective health (chronic conditions). Age moderated some of these effects, suggested that these associations may be strongest among older adults.

